# Complete Genome Sequence of *Mesorhizobium ciceri* bv. biserrulae WSM1497, an Efficient Nitrogen-Fixing Microsymbiont of the Forage Legume *Biserrula pelecinus*

**DOI:** 10.1128/genomeA.00902-17

**Published:** 2017-08-31

**Authors:** Rachel J. M. Brewer, Timothy L. Haskett, Joshua P. Ramsay, Graham W. O’Hara, Jason J. Terpolilli

**Affiliations:** a Centre for Rhizobium Studies, School of Veterinary and Life Sciences, Murdoch University, Perth, Australia; b School of Biomedical Sciences and Curtin Health Innovation Research Institute, Curtin University, Perth, Australia

## Abstract

We report here the complete genome sequence of *Mesorhizobium ciceri* bv. biserrulae strain WSM1497, the efficient nitrogen-fixing microsymbiont and commercial inoculant in Australia of the forage legume *Biserrula pelecinus*. The genome consists of 7.2 Mb distributed across a single chromosome (6.67 Mb) and a single plasmid (0.53 Mb).

## GENOME ANNOUNCEMENT

The reduction (or fixation) of atmospheric nitrogen into ammonia by soil bacteria (rhizobia) in symbiosis with legumes is critical to global nitrogen cycling and sustainable agriculture (1, 2). Nitrogen-fixing symbioses are established when rhizobia infect legume roots, resulting in the formation of root nodules (3, 4). Rhizobia in the genus *Mesorhizobium* are known to harbor genes essential to nodule development (*nod*) and nitrogen fixation (*nif* and *fix*) on mobile chromosomal regions referred to as symbiosis integrative and conjugative elements (ICEs) (5). Symbiosis ICEs may comprise a single contiguous region of ∼500 kb of DNA, such as in *Mesorhizobium loti* R7A (6) and *Mesorhizobium ciceri* CC1192 (7), or may be structurally more complex, such as the recently identified tripartite symbiosis ICEs in *Mesorhizobium ciceri* bv. biserrulae strains WSM1271 and WSM1284 (8, 9).

*M. ciceri* bv. biserrulae strain WSM1497 is the commercial inoculant in Australia for the forage legume *Biserrula pelecinus* (10, 11). Although *B. pelecinus*-nodulating rhizobia were initially absent in Australian soils, indigenous soil bacteria have since acquired symbiosis genes from WSM1497, resulting in the evolution of novel *Biserrula pelecinus*-nodulating strains, which fix nitrogen suboptimally on this host (12). The draft genome sequence data suggested that WSM1497 harbors a mobile tripartite symbiosis ICE (7). The availability of the full-genome sequence of WSM1497 will therefore enable investigation into horizontal gene transfer of symbiosis genes from this strain to soil rhizobia.

WSM1497 genomic DNA was extracted and purified from a tryptone-yeast-grown culture (13) using a DNeasy blood and tissue kit (catalog no. 69504; Qiagen). Whole-genome sequencing was performed using both Pacific Biosciences (PacBio) single-molecule real-time sequencing and Illumina HiSeq 2500 technology by Macrogen (South Korea). PacBio sequencing generated 136,085 postfilter subreads, with an average length of 4,057 bp (∼77-fold depth of coverage). Illumina HiSeq sequencing was used to generate 25,226,358 101-bp paired-end reads (∼354-fold depth of coverage). Illumina adaptors were removed using nesoni:clip (https://github.com/Victorian-Bioinformatics-Consortium/nesoni). Filtered Illumina and PacBio reads were used to generate a hybrid *de novo* assembly using SPAdes version 3.10.0 (14), producing two large circular contigs that were annotated using the NCBI Prokaryotic Genome Annotation Pipeline (PGAP) (http://www.ncbi.nlm.nih.gov/genomes/static/Pipeline.html). The genome is 7,198,121 bp in length and has an average GC content of 62.4%. There are 7,006 coding sequences that are distributed across a single circular chromosome of 6,666,492 bp and a single plasmid (pWSM1497) of 531,629 bp.

Our preliminary analysis of the complete WSM1497 genome indicates that it harbors a tripartite symbiosis ICE (ICE*Mc*Sym^1497^), delineated by three pairs of integrase attachment sites similar to those of WSM1271 (9). The total size of ICE*Mc*Sym^1497^ is 468.3 kb, which comprises the separate regions α (bp 6100975 to 6544486), β (bp 2746886 to 2766245), and γ (bp 2527429 to 2532841). Region α harbors symbiosis genes and biotin and nicotinate biosynthetic clusters similar to those found on other symbiosis ICEs. ICE*Mc*Sym^1497^ also encodes a conjugative type IV secretion system and contains homologs of quorum-sensing genes known to regulate ICE*Ml*Sym^R7A^ excision and transfer in *M. loti* R7A (6, 15, 16). Work is under way to investigate the mobility of ICE*Mc*Sym^1497^.

### Accession number(s).

The nucleotide sequence of the complete genome of WSM1497 has been deposited in GenBank under the accession numbers CP021070 (chromosome) and CP021071 (plasmid pWSM1497).
